# Molecularly Imprinted Polymers as Biomimetic Test Zones in Paper-Based Nucleic Acid Assays—Comparing Vertical and Lateral Flow Formats

**DOI:** 10.3390/bios16030175

**Published:** 2026-03-21

**Authors:** Jennifer Marfà, Anaixis del Valle, Maria Del Pilar Taboada Sotomayor, María Isabel Pividori

**Affiliations:** 1 Grup de Sensors i Biosensors, Departament de Química, Universitat Autònoma de Barcelona, 08193 Bellaterra, Spain; jennifer.marfa@uab.cat (J.M.); anaixis.delvalle@uab.cat (A.d.V.); 2 Biosensing and Bioanalysis Group, Institute of Biotechnology and Biomedicine, Universitat Autònoma de Barcelona, 08193 Bellaterra, Spain; 3 Department of Analytical Chemistry, Institute of Chemistry, São Paulo State University (UNESP), Araraquara 14800-060, SP, Brazil; m.sotomayor@unesp.br

**Keywords:** molecularly imprinted polymers, paper-based diagnostic platforms, biomimetic receptors, nucleic acid vertical flow assay

## Abstract

The development of rapid and sensitive point-of-care nucleic acid tests benefits from robust synthetic recognition elements. Here, a biotin-specific molecularly imprinted polymer (MIP) was synthesized using an optimized protocol and integrated as a biomimetic test zone into two paper-based formats: nucleic acid vertical flow (NAVF) and nucleic acid lateral flow (NALF). Both platforms were evaluated for the detection of double-tagged PCR amplicons from *Escherichia coli*. NAVF enabled a 3 min visual readout with an LOD of 1.00 × 10^−2^ ng mL^−1^. NALF provided a total assay time of <15 min and achieved a visual LOD of 3.17 × 10^−2^ ng mL^−1^. Overall, the results demonstrate the versatility of biotin-MIPs as stable synthetic receptors for rapid, low-cost paper-based nucleic acid assays, with NAVF prioritizing speed and design flexibility and NALF prioritizing higher analytical sensitivity.

## 1. Introduction

The global healthcare landscape is evolving rapidly, driven by the growing demand for rapid, sensitive, and accessible diagnostic technologies [1,2]. Point-of-care (POC) tests have become essential tools due to their ability to provide immediate results at or near the site of patient care, thereby reducing reliance on centralized laboratories [3,4]. Recent global health crises, including tuberculosis, HIV, and the COVID-19 pandemic, have further highlighted the importance of rapid testing to support early clinical decision-making, control disease transmission, and ensure that appropriate treatment is administered after a confirmed diagnosis [5,6,7,8].

Among diagnostic devices, paper-based platforms have gained significant attention due to their low cost, ease of use, portability, and minimal infrastructure requirements [9,10]. Lateral flow assays (LFAs), best known from home pregnancy tests and SARS-CoV-2 antigen tests, are one of the most well-established paper-based formats. LFAs rely on capillary action to transport liquid samples along a porous membrane, where the target analytes are selectively captured by immobilized recognition elements, producing a signal that can be read by the naked eye or quantified using optical readers (e.g., portable scanners or smartphone-based systems) [11,12,13]. Their ability to detect a wide range of analytes, including proteins, nucleic acids, and small molecules, makes them particularly valuable in decentralized and resource-limited settings [14,15,16,17]. However, LFAs have inherent limitations that restrict their broader applicability, particularly when multiplexing capabilities or larger sample volumes are required [18,19]. In addition, LFAs are susceptible to the hook effect, in which excess concentration of target analyte saturates the capture molecules, reducing signal intensity and potentially leading to false-negative results [20,21].

Vertical flow assays (VFAs) have emerged as an alternative non-conventional format that addresses many of these limitations [22,23,24]. Although based on similar principles, VFAs use gravity, capillary forces, and, in some cases, external forces to drive the sample vertically through stacked porous membranes [25]. This configuration offers several advantages over LFAs, including shorter assay times, the ability to process larger sample volumes, and reduced susceptibility to the hook effect [22]. Moreover, VFAs have demonstrated improved multiplexing capabilities, allowing the simultaneous detection of multiple analytes in a single device [26,27,28,29].

Despite their usefulness in diverse diagnostic scenarios, both LFAs and VFAs still rely heavily on biological recognition elements, typically antibodies, which may limit their stability and applicability in resource-limited environments where refrigeration and cold chain logistics are not available. Molecularly imprinted polymers (MIPs) have therefore been proposed as synthetic alternatives to overcome these constraints [30]. MIPs are prepared by polymerizing functional monomers around a template molecule, which is later removed to leave specific binding cavities tailored to the target analyte [31,32]. Compared to biological receptors, MIPs exhibit superior chemical and mechanical stability, extended shelf life, and can be adapted to recognize a wide range of target molecules, making them promising candidates for the development of reliable and cost-effective paper-based diagnostic devices [33,34].

Molecular imprinting technology (MIT) generates synthetic receptors by polymerizing functional monomers and crosslinkers around a template, followed by template removal to create binding cavities that are complementary in size, shape and functional-group distribution. Recent state-of-the-art work shows that the field is rapidly moving beyond conventional bulk MIPs toward engineered architectures that improve binding-site accessibility, accelerate mass transport and increase robustness in complex matrices. In particular, molecularly imprinted framework materials combine the selectivity of imprinting with the high surface area and tunable porosity of framework scaffolds, enabling better transport properties and more efficient adsorption/recognition [35]. In parallel, the evolution toward nanoscale MIPs (nanoMIPs) and biomedical implementations emphasizes advanced fabrication strategies and fast binding kinetics, reinforcing the importance of rational design to obtain stable, high-performance recognition elements under realistic operating conditions [36]. Beyond these advances, MIPs are increasingly positioned as practical antibody mimics in bioanalysis, where rational control over monomer–template interactions and polymer format can be used to tune recognition, robustness, and performance in complex samples [37]. In parallel, recent tutorial and perspective articles have consolidated key design and validation principles for chemical sensing and biomarker detection, while highlighting the added value of direct nanoscale integration of MIPs with transducers to improve mass transport and signal transduction in high-performance sensing interfaces [38,39,40]. These developments provide a framework for positioning the present study, where a biotin-specific MIP is implemented as a biomimetic capture zone in paper-based nucleic-acid vertical and lateral flow formats to achieve rapid, robust and low-cost detection of doubly labeled PCR amplicons.

This work builds upon a previously developed and validated biotin-specific molecularly imprinted polymer (biotin-MIP) [41,42] and explores its integration as a synthetic recognition element into nucleic acid lateral flow (NALF) and nucleic acid vertical flow (NAVF) platforms for the detection of double-tagged PCR amplicons from Escherichia coli. After optimization of the components of the NAVF device to improve overall sensitivity, the analytical performance of both non-conventional and conventional formats was evaluated and compared. By exploring the unique properties of MIPs and combining them with the complementary advantages of NAVF and NALF platforms, this study aims to contribute to the development of POC diagnostics that are sensitive, robust, and adaptable to different application needs.

## 2. Materials and Methods

### 2.1. Instrumentation and Materials

Scanning electron microscopy (SEM) images were obtained using an EVO MA 10 microscope (Carl Zeiss, Jena, Germany) operating at an acceleration voltage of 3.0 kV. Fourier transform infrared (FT-IR) spectra were recorded in the range of 4000–400 cm^−1^ with a Bruker ALPHA II spectrometer (Bruker Optics GmbH, Ettlingen, Germany) equipped with a platinum attenuated total reflection (ATR) accessory. MIP suspensions were prepared using a Sonics Vibra-Cell ultrasonic processor (VCX 130PB, 130 W) equipped with a 3 mm stepped microtip probe (No. 630-0422, Sonics & Materials Inc., Newtown, CT, USA). The polymerase chain reaction (PCR) was carried out in a SimpliAmp Thermal Cycler (Applied Biosystems, Foster City, CA, USA).

The lateral flow strips were assembled using adhesive backing cards (No. KN-2211, Kenosha B.V., Amstelveen, The Netherlands) and cellulose fiber sample pads SureWick C083 (No. CFSP203000, Merck Millipore, Darmstadt, Germany). The FF80HP nitrocellulose membrane (No. 10547002) and the CF7 absorbent pad (No. 8117-2250) were obtained from Cytiva (Marlborough, MA, USA). The cartridges for the nucleic acid vertical flow assay (NAVF) devices were purchased from MedMira Inc. (No. VF-1-01, Halifax, NS, Canada). All materials and components used in the design and optimization of the NAVF platform are listed in Section S1 (Supplementary Information).

### 2.2. Chemicals and Biochemicals

The reagents used for the synthesis of the biotin-MIP included biotin (No. B4501), acrylic acid (No. 147230), ethylene glycol dimethacrylate (EGDMA, No. 335681), and 2,2′-azobisisobutyronitrile (AIBN, No. 441090), all purchased from Sigma-Aldrich (St. Louis, MO, USA). Ethanol absolute (No. 1.00983), methanol (No. 8.22283), and acetic acid (No. 45754) were supplied by VWR. Prior to polymerization, stabilizers and inhibitors were removed from the functional monomer and the cross-linker by passing the solutions through a basic alumina column. Tris(hydroxymethyl)aminomethane (TRIS, No. T6066), Tween 20 (No. P7949), sodium chloride (No. S3014), polyethylene glycol 4000 (PEG 4000, No. 81240), anti-digoxigenin POD Fab fragments (antiDIG-HRP, No. 11207733910, Roche Diagnostics), and 3,3′,5,5′-tetramethylbenzidine (TMB, liquid substrate system for membranes, No. T0565) were obtained from Sigma-Aldrich. All solutions were prepared using Milli-Q water and analytical-grade reagents. The composition of the buffers was as follows: TRIS-T (0.1 M TRIS, 0.15 M NaCl, 0.05% Tween 20, pH 7.4) and TRIS (0.1 M TRIS, 0.15 M NaCl, pH 7.4). The primers for the double-tagging PCR of *E. coli* were purchased from Sigma-Aldrich, and their sequences are detailed in Table S1 (Supplementary Information).

### 2.3. Synthesis and Characterization of Molecularly Imprinted Polymers for Biotin

The biotin-MIP was synthesized by precipitation polymerization following a previously reported method with minor modifications [41]. Briefly, biotin (0.2 mmol) and acrylic acid (0.8 mmol) were dissolved in 80 mL of an ethanol–water mixture (70:30 *v*/*v*) and allowed to interact for 4 h at room temperature. Afterward, EGDMA (4.0 mmol) and AIBN (0.05 mmol) were added, and the system was purged with N_2_ for 20 min. The reaction mixture was then heated at 60 °C for 18 h under a nitrogen atmosphere. After polymerization, the biotin-MIP particles were collected by filtration and dried at 50 °C for 5 h. The template and unreacted monomers were removed by Soxhlet extraction with methanol–acetic acid (90:10 *v*/*v*), followed by methanol and then water. The non-imprinted polymer (NIP) was prepared following the same procedure, but without the addition of the template.

The biotin-MIP and NIP were characterized by FT-IR to identify the functional groups of the polymers and confirm the complete removal of the biotin template, and by SEM to assess their surface morphology. For SEM analysis, conductive carbon tape was applied to aluminum stubs, and a small amount of each polymer was evenly spread on the surface. The samples were then sputter-coated with an Au–Pd alloy (80:20) for 4 min, depositing a 15–20 nm thick conductive layer. The particle size distribution was determined by measuring 400 particles from different SEM images.

### 2.4. Assembly of Vertical Flow Cartridges, Lateral Flow Strips, and Integration of Molecularly Imprinted Polymers for Biotin into the Membrane

**Figure 1:**
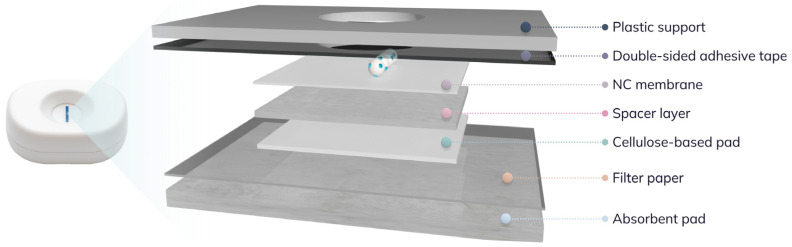
Schematic representation of the components used in the final design of the NAVF cartridges. From top to bottom, the assembly consist of (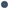) a plastic support with a central hole for sample addition; (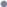) a double-sided adhesive tape to seal the central opening and to secure all the layers; (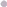) a NC membrane where the biotin-MIP is immobilized; (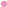) a spacer layer to reduce sample retention and maintain the separation between the layers; (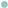) a cellulose-based pad to minimize non-specific background; (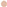) a filter paper to reinforce the structural stability and hold all stacked layers correctly assembled; and (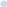) an absorbent pad at the bottom to collect excess reagents and prevent backflow.

After optimization of all device components, the NAVF cartridges were assembled by sequentially stacking the selected layers in a defined order from top to bottom, as shown in Figure 1. Further information on the components used in the final design and the detailed assembly procedure is provided in Section S4 (Supplementary Information).

For the NAVF device, 5 µL of the biotin-MIP suspension (10 mg mL^−1^) was manually applied to the reaction zone using a micropipette tip. The cartridges were then dried at 39 °C for 2 h and stored overnight in a desiccator. To reduce background signal and prevent non-specific adsorption, 40 µL of a 0.2% (*w*/*v*) PEG 4000 blocking solution was added to the reaction zone and dried again at 39 °C for 3 h.

For the preparation of NALF strips, all components were assembled on an adhesive backing card, with the sample and absorbent pads overlapping the NC membrane by 2 mm. The selection of FF80HP for the MIP-based NALF was based on a previous membrane optimization study, which demonstrated that this backed nitrocellulose membrane provides the most suitable performance for this MIP-based lateral-flow configuration [42].

Once assembled, the strips were cut to a width of 8 mm using a guillotine. The integration of the biotin-MIP into the NC membrane was performed following the same procedure described above. The NC membrane was then blocked by adding 5 µL of 0.2% (*w*/*v*) PEG 4000, and the strips were dried at 39 °C for 1.5 h. To maintain consistent assay performance, the paper-based devices and related materials were protected from humidity and stored under dry conditions before use.

The integration and distribution of the biotin-MIP within the NC membrane were assessed by SEM at an acceleration voltage of 3.0 kV. Since the NC membranes used in both assay formats have similar properties and pore size, the FF80HP was selected as the representative substrate for this study.

### 2.5. Optimization of the Integration of the Molecularly Imprinted Polymers in Vertical Flow Cartridges

The components used in the construction of the NAVF platform shown in Figure 1 were optimized to improve the sensitivity of the assay and to minimize non-specific background signals. All optimization studies were performed according to the procedure described in Section S4 (Supplementary Information), where only the component evaluated was varied, while all other parameters were kept constant. The main components studied and optimized included NC membranes, cellulose-based pads, absorbent pads, spacer layers, double-sided adhesive tapes, and filter paper.

Several NC membranes were tested, including unbacked membranes with different capillary flow rates (AE98, AE99, AE100), as well as Protran membranes BA85 (0.45 µm) and BA83 (0.2 µm), which are commonly used in blotting applications. Cellulose-based pads (C083 and CF4) and absorbent pads (CF5 and CF7) were evaluated to ensure uniform liquid distribution within the reaction zone, provide efficient liquid absorption, maintain unidirectional flow, and prevent the backflow of reagents or samples.

To ensure proper alignment and immobilization of all stacked membranes, several medical-grade and microfluidic double-sided adhesive tapes (1510, 1522, 1567, 9965, and 9969) were assessed for water resistance, sealing strength, and capacity to maintain the integrity of biological samples without causing contamination. Finally, filter papers of various thicknesses were tested to improve the structural integrity of the platform and to ensure that all layers were correctly assembled during the assay. Further experimental details, including optimization procedures and test conditions, are provided in Section S4 (Supplementary Information).

### 2.6. Detection of Double-Tagged Amplicons by Nucleic Acid Vertical Flow Assay

The amplification and double-tagging of the gDNA extracted from *E. coli* were performed using forward and reverse primers labeled at the 5′ end with biotin (BIO) and digoxigenin (DIG), respectively [43]. Detailed information on the *E. coli* strain, bacterial growth, DNA extraction, and PCR protocol can be found in Section S2 (Supplementary Information).

**Figure 2:**
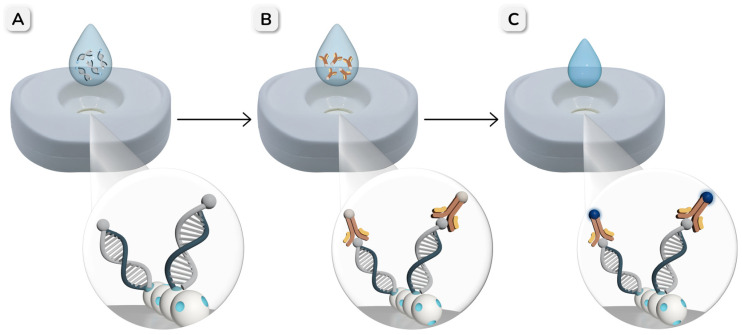
Schematic representation of the procedure for the detection of double-tagged PCR amplicons of *E. coli*, using the biotin-MIP integrated in the reaction zone of the NAVF platform. (**A**) The sample is added to the reaction zone, where the biotin-MIP specifically captures the BIO tag on the amplicons. (**B**) Then, antiDIG-HRP is applied and binds to the DIG tag on the captured amplicons. (**C**) Finally, the TMB substrate is added to generate a colorimetric signal that can be visually evaluated or quantified by image analysis.

The procedure for the detection of double-tagged amplicons of *E. coli* using the NAVF platform is schematically illustrated in Figure 2. Before sample application, 50 µL of TRIS buffer was added to the reaction zone to rehydrate the membranes. Subsequently, 50 µL of different concentrations of the amplicons was applied and allowed to be completely absorbed through the stacked membranes. During this step, the biotin-MIP immobilized on the NC membrane selectively captured the BIO tag on the amplicons. Then, 50 µL of antiDIG-HRP (0.5 U mL^−1^) was added to enable optical detection through its interaction with the DIG tag present on the captured amplicons. After two washing steps with 50 µL of TRIS-T buffer, 50 µL of TMB substrate was added to the reaction zone to produce a colorimetric signal, and the results were visually assessed after 3 min. For semi-quantitative analysis, the images of the cartridges were captured using a Xiaomi Mi 11T smartphone and processed with ImageJ software version v1.54p.

### 2.7. Detection of Double-Tagged Amplicons by Nucleic Acid Lateral Flow Assay

**Figure 3:**
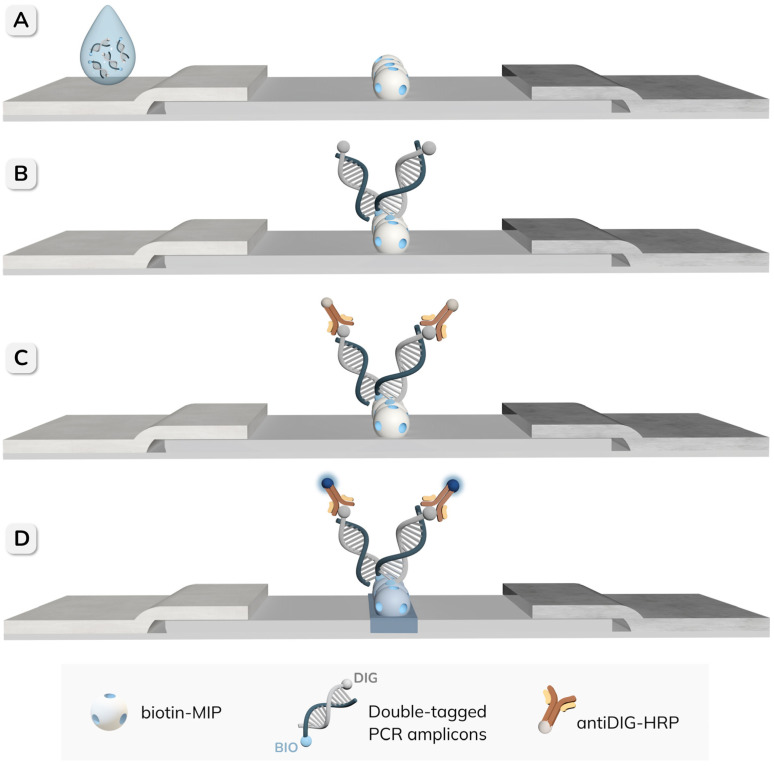
Schematic representation of the NALF procedure for the detection of double-tagged PCR amplicons using the biotin-MIP as a test line. (**A**) A sample containing double-tagged amplicons is applied to the sample pad. (**B**) As the sample migrates along the strip, the biotin-MIP selectively captures the BIO tag on the amplicons. (**C**) AntiDIG-HRP is then added to enable optical detection through its interaction with the DIG tag on the captured amplicons. (**D**) After the addition of the TMB substrate, the appearance of a blue color indicates a positive result, whereas the absence of color corresponds to a negative result. The resulting signal can be interpreted visually or analyzed semi-quantitatively using ImageJ software.

For comparison, the detection of the same double-tagged amplicons was also performed by NALF, as shown in Figure 3. In this case, 50 µL of serial dilutions of the amplicons were applied to the sample pad and allowed to migrate along the strip. After 5 min, 50 µL of TRIS buffer was added to facilitate the complete migration of the amplicons to the absorbent pad. Following the addition of 50 µL of antiDIG-HRP (0.5 U mL^−1^), the strips were washed twice with 50 µL of TRIS-T and once with 50 µL of TRIS buffer. Finally, 5 µL of TMB substrate was added, and the results were evaluated after 10 min, either visually or by quantifying the intensity of the test line using ImageJ software.

### 2.8. Data Interpretation and Statistical Analysis

To ensure consistency and avoid luminosity bias, all cartridges and strips were photographed under identical conditions in a portable photo studio equipped with two 1500 lm LED spotlights and a color temperature of 6500 K. The images were taken from a fixed distance of 35 cm using the rear camera of a Xiaomi Mi 11T with a resolution of 180 megapixels. During image acquisition, autofocus was enabled, and the camera flash was turned off. For quantitative analysis, the images were opened in ImageJ and converted to 8-bit grayscale. The rectangular selection tool was used to define the region of interest (ROI), and the area under each peak, corresponding to the relative signal intensity, was numerically integrated using the Gel Analysis toolbox in ImageJ. All assays were performed in triplicate. Statistical analyses were carried out with Prism v10.2.2 (GraphPad, San Diego, CA, USA). To determine the sensitivity and specificity of the assays, the signal intensity data obtained by image processing were fitted by non-linear regression using a four-parameter logistic (4PL) model.

### 2.9. Safety Considerations

All procedures involving the handling of *E. coli* were conducted in a Biosafety Level 2 (BSL-2) laboratory under containment conditions. Experimental work was carried out in a certified biosafety cabinet. All biological materials and waste generated during the experiments were decontaminated by autoclaving or chemical disinfection before being discarded, in accordance with U.S. Department of Health and Human Services BSL-2 guidelines.

## 3. Results and Discussion

### 3.1. Synthesis and Characterization of Molecularly Imprinted Polymers for Biotin

Figure S1 (Supplementary Information) shows the FT-IR spectra of the biotin-MIP, before and after template removal, together with the corresponding NIP.

A weak peak at 1637 cm^−1^, attributed to the C=C stretching of vinyl groups, was observed in all spectra with comparable intensity, suggesting a minimal presence of unreacted double bonds and a similar degree of polymerization. Additional peaks corresponding to C-H stretching vibrations of methylene and methyl groups in the aliphatic polymer backbone, C=O stretching of carbonyl groups, and C-O-C stretching of ester groups confirmed the formation of the polymer network. After template removal, the broad band between 3600 and 3400 cm^−1^ shifted toward higher wavenumbers, which is consistent with the disruption of the hydrogen bonds between the template and the functional monomer. Importantly, the close similarity between the spectra of the biotin-MIP after template removal and the NIP confirmed the complete removal of the biotin template. A more detailed discussion of the FT-IR results, including peak assignments, is provided in Section S3 (Supplementary Information).

**Figure 4:**
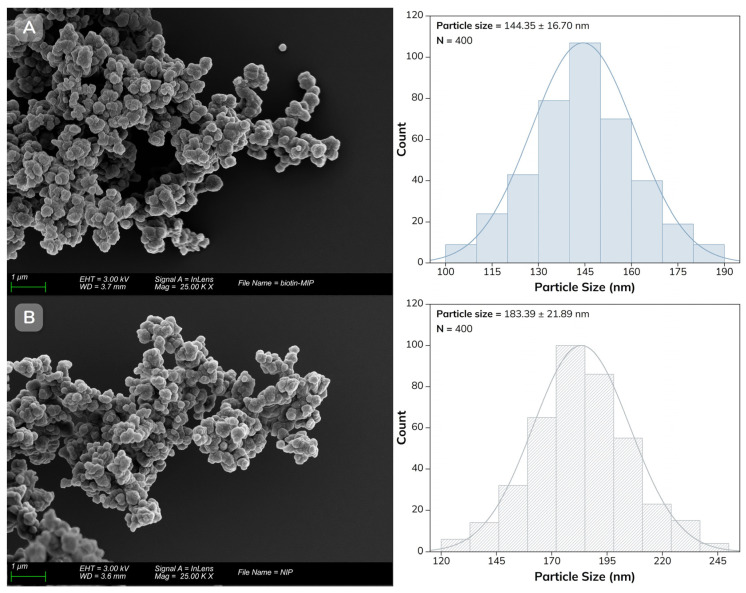
SEM images and corresponding particle size distributions of (**A**) biotin-MIP and (**B**) NIP. Images were acquired at 25,000× magnification (scale bars: 1 µm). Particle sizes were determined by measuring 400 particles per sample.

The surface morphology and particle size distribution of the polymers were then characterized by SEM. As shown in Figure 4A,B, the biotin-MIP and NIP are irregular spherical nanoparticles that appear agglomerated. Further analysis of the SEM images showed that the average particle size of the biotin-MIP was 144.3 nm (SD = 16.7 nm). In contrast, NIP particles were larger and more irregular, with an average size of 183.4 nm (SD = 21.9 nm) and a broader size distribution. Although no other significant morphological differences were observed, these results suggest that the presence of the template during the imprinting process may affect the particle formation.

From the specific binding isotherm (one-site binding model) performed in previous studies [42], the dissociation constant and maximum binding capacity of the biotin-MIP were determined as Kd = 7.09 × 10^−10^ mol L^−1^ and Bmax = 304 fmol mg^−1^, respectively, using biotin-HRP as a model biotinylated ligand. While the biotin–streptavidin pair remains the gold standard in intrinsic affinity (Kd ≈ 10^−15^ mol L^−1^), these values confirm high-affinity, imprinting-driven recognition and provide quantitative support for the use of the biotin-MIP as a robust solid capture phase integrated into paper-based test zones [42].

### 3.2. Assembly of Vertical Flow Cartridges, Lateral Flow Strips, and Integration of Molecularly Imprinted Polymers for Biotin into the Membrane

**Figure 5:**
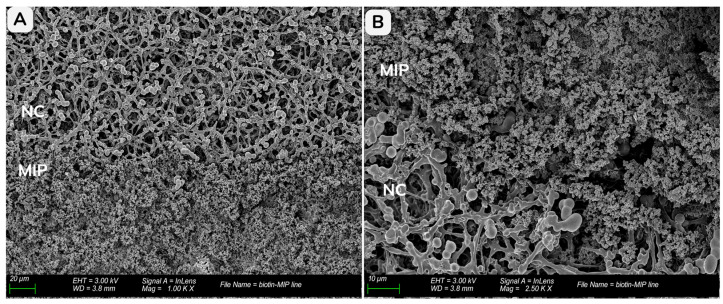
SEM images of the biotin-MIP (MIP) integrated into the FF80HP nitrocellulose (NC) membrane (pore size = 5 µm). (**A**) View of the interface region between the biotin-MIP layer and the NC membrane at 1000× magnification; the lower half corresponds to the biotin-MIP impregnated region, whereas the upper half corresponds to the bare FF80HP nitrocellulose structure. (**B**) Higher-magnification view (2500×) showing biotin-MIP particles distributed within the porous NC structure in the interface area.

Figure 5 presents SEM micrographs of the FF80HP nitrocellulose (NC) membrane (pore size = 5 µm) after integration of the biotin-MIP test zone, providing a direct visualization of how the polymer is accommodated within the porous support. At lower magnification (Figure 5A, 1000×), two clearly differentiated regions can be observed within the same field of view: the lower half corresponds to the MIP-impregnated zone, characterized by a dense granular texture, whereas the upper half preserves the characteristic fibrous network of the bare nitrocellulose. The sharp transition between both morphologies confirms the formation of a well-defined MIP test area rather than a diffuse spreading of material across the membrane. At higher magnification (Figure 5B, 2500×), the granular biotin-MIP is seen intimately associated with the NC matrix, with particles distributed within the pore network, supporting the notion that the small particle size enables penetration and anchoring throughout the three-dimensional porous structure. Overall, these images confirm successful immobilization of the biotin-MIP to the membrane and indicate that the deposition process preserves the integrity of the nitrocellulose scaffold, which is essential to maintain consistent capillary transport during assay operation.

### 3.3. Optimization of the Integration of Molecularly Imprinted Polymers in Vertical Flow Cartridges

The NALF strip configuration, including membrane selection, was previously optimized in an earlier work [38], where the backed nitrocellulose FF80HP was identified as the most suitable membrane for this MIP-based lateral-flow format. However, this backed membrane cannot be transferred to the NAVF configuration because the polyester support layer is essentially impermeable to through-thickness flow, which prevents the required vertical permeation across the membrane and disrupts the downwards transport through the stacked layers of the cartridge. For this reason, the main components of the NAVF platform were independently optimized, including the NC membrane, spacer layer, cellulose-based and absorbent pads, adhesives, and filter papers. All optimization studies were performed in triplicate, and the corresponding experimental details are provided in Section S4 (Supplementary Information). Among the NC membranes tested, AE98 showed the highest signal intensity and the lowest background, closely followed by AE100 (Figure S3, Supplementary Information), and it also enabled successful immobilization of the biotin-MIP, confirming its suitability for NAVF. Importantly, the pore size (5 µm) and flow rate (160–210 s/4 cm) of AE98 were similar to those of the FF80HP membrane used in the NALF platform. Therefore, AE98 was selected for further studies, and the specifications of all assessed NC membranes are provided in Table S3 (Supplementary Information).

The use of a VF2 pad as a spacer layer beneath the reaction membrane was also evaluated. Compared to cotton-based materials, this glass fiber pad promoted a faster flow through the stacked layers and significantly reduced sample retention, resulting in a lower background signal (Figure S5, Supplementary Information). Moreover, it prevented direct contact between the NC membrane and the absorbent layer, which could otherwise cause interference or background noise. The properties of VF2 are summarized in Table S4 (Supplementary Information). Further optimization of the cellulose-based pads revealed that CF4 was the most appropriate due to its high absorption capacity and low protein-binding properties, which reduced the non-specific background signal and increased the sensitivity of the assay. The comparative results obtained for C083 and CF4 are shown in Figure S6 (Supplementary Information), and their specifications are provided in Table S5 (Supplementary Information). CF7 was then selected as the absorbent layer for its capacity to handle large liquid volumes, thus preventing backflow and reducing the risk of reagent pooling in the reaction zone. Further information on the absorbent layers is provided in Table S6 (Supplementary Information).

To ensure proper immobilization of the layers and promote downward flow, several double-sided adhesive tapes were studied. As shown in Figure S7 (Supplementary Information), the 1567 tape provided a robust, water-resistant seal that prevented leakage and ensured consistent unidirectional flow. Finally, filter papers were assessed to identify materials that could reinforce the structural integrity of the platform and hold the layers together. Figure S8 (Supplementary Information) shows that the use of thin filter paper (12.5 g m^−2^) provided additional support without affecting the flow rate, facilitated correct membrane alignment, and contributed to the overall functionality and reliability of the assay.

### 3.4. Detection of Double-Tagged Amplicons by Nucleic Acid Vertical Flow Assay

**Figure 6:**
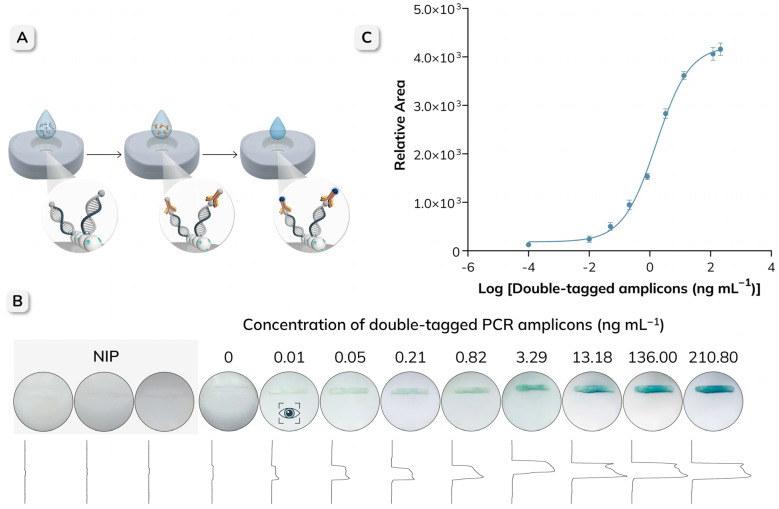
(**A**) Schematic representation of the procedure for the detection of double-tagged PCR amplicons of *E. coli* using the biotin-MIP integrated into the reaction zone of the NAVF platform. (**B**) Representative NAVF results for increasing concentrations of double-tagged PCR amplicons (0–210.8 ng mL−1); the corresponding relative area values are shown below each concentration. The eye icon indicates the visual limit of detection (vLOD = 0.01 ng mL^−1^). The PCR negative control (no target DNA, therefore no amplicon) is included as a blank. NIP (non-imprinted polymer) strips evaluated at 210 ng mL^−1^ are included as a control to assess non-specific binding. (**C**) Calibration curve obtained by plotting relative area values as a function of the logarithm of the amplicon concentration. Error bars represent the standard deviation (n = 3), and the individual replicates are provided in Figure S9 (Supplementary Information).

The procedure for the detection of double-tagged PCR amplicons of *E. coli* by NAVF is shown schematically in Figure 6A. The performance of the optimized platform was evaluated using different concentrations of the amplicons, ranging from 0 to 210.80 ng mL^−1^. It is important to note that the readout of the results can be achieved in just 3 min. As shown in Figure 6B, the intensity of the blue color in the test line increased with the concentration of the amplicons, while the PCR negative control showed no visible signal, indicating negligible non-specific adsorption on the membrane or the biotin-MIP. The images of all triplicate NAVF cartridges and their respective peak areas are shown in Figure S9 (Supplementary Information). NIP (non-imprinted polymer) strips evaluated at maximun concentration (210 ng mL^−1^) are included as a control to assess non-specific binding and to confirm the contribution of molecular imprinting to target capture. The lowest concentration at which a positive signal was still visible was 0.01 ng mL^−1^ and was therefore considered the visual limit of detection (vLOD).

For further analysis, the line intensities were quantified by processing the images of the NAVF cartridges at different concentrations. The area under each peak was numerically integrated using the Gel Analysis toolbox in ImageJ, and the resulting values were fitted using a non-linear regression (Sigmoidal 4PL, GraphPad Prism 11.0.0, R^2^ = 0.9957) (Figure 6C). The limit of detection (LOD) was estimated by analyzing the negative PCR control (n = 3), which contained all the reagents except the target DNA. The cut-off value was calculated as the mean signal of the negative controls plus three times the standard deviation (SD), and then interpolated into the calibration curve, resulting in an LOD of 2.53 × 10^−2^ ng mL^−1^. Similarly, the limit of quantification (LOQ), defined as the mean negative control signal plus ten times the SD, was determined to be 2.76 × 10^−2^ ng mL^−1^. These results demonstrate that the NAVF platform can reliably detect low concentrations of the analyte, highlighting its potential for applications requiring rapid and accurate detection.

### 3.5. Detection of Double-Tagged Amplicons by Nucleic Acid Lateral Flow Assay

**Figure 7:**
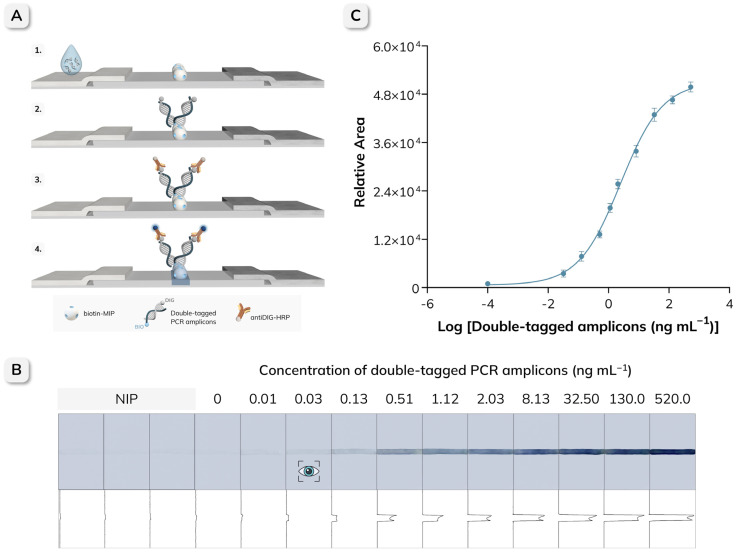
(**A**) Schematic representation of the NALF assay for the detection of double-tagged PCR amplicons of *E. coli* using the biotin-MIP as a test line. (**B**) Representative images of NALF strips obtained for increasing concentrations of double-tagged PCR amplicons (0–520 ng mL−1); the corresponding relative area values, calculated from ImageJ-processed images, are shown below each strip. The eye icon indicates the visual limit of detection (vLOD = 0.03 ng mL^−1^). The PCR negative control (no target DNA, therefore no amplicon) is included as a blank. NIP (non-imprinted polymer) strips evaluated at maximum concentration (520 ng mL^−1^) are included as a control to assess non-specific binding and to confirm the contribution of molecular imprinting to target capture. (**C**) Calibration curve obtained by plotting relative area values as a function of the logarithm of the amplicon concentration; negative controls are also included. Error bars represent the standard deviation (n = 3), and the individual replicates are provided in Figure S10 (Supplementary Information).

The detection of double-tagged PCR amplicons of *E. coli* was also performed using the NALF platform, as schematically illustrated in Figure 7A. Serial dilutions of the amplicons, ranging from 0 to 520 ng mL^−1^, were evaluated, and the results are shown in Figure 7B. All NALF strip images obtained in triplicate and their corresponding relative peak areas are provided in Figure S10 (Supplementary Information). NIP (non-imprinted polymer) strips evaluated at maximum concentration (520 ng mL^−1^) are included as a control to assess non-specific binding and to confirm the contribution of molecular imprinting to target capture. In this case, the total assay time, from sample addition to strip readout, was less than 15 min. As expected, the signal intensity increased in proportion to the concentration of the amplicons, whereas the strips containing the NIP and those corresponding to the negative PCR control showed no visible signal, confirming the sensitivity and specificity of the assay. Based on the lowest positive signal detectable by the naked eye, the vLOD was determined to be 3.17 × 10^−2^ ng mL^−1^. For quantitative evaluation, the images of the NALF strips were then processed as described above, and the resulting data were fitted using a non-linear regression (Sigmoidal 4PL, GraphPad Prism, R^2^ = 0.9976) (Figure 7). The LOD and LOQ obtained were 2.95 × 10^−3^ ng mL^−1^ and 4.02 × 10^−3^ ng mL^−1^, respectively.

A comparison of the two platforms revealed that the NAVF provided a lower vLOD (0.01 ng mL^−1^) than NALF (0.03 ng mL^−1^), indicating higher sensitivity for visual detection. However, when analyzed quantitatively, NALF achieved a significantly lower LOD (2.95 × 10^−3^ ng mL^−1^) compared to NAVF (2.53 × 10^−2^ ng mL^−1^), corresponding to an approximately one order of magnitude improvement. This increased sensitivity is primarily attributed to the design of the NALF platform, which improves the binding efficiency by allowing for a longer interaction time between the amplicons and the biotin-MIP. Despite these differences, the successful integration of the biotin-MIP as a synthetic recognition element into both platforms demonstrates its versatility and the excellent analytical performance for the targeted detection of biotinylated amplicons. Each platform offers distinct advantages that can be useful in different diagnostic scenarios, and the choice between them should therefore be guided by the specific needs of the intended application. Accordingly, the NAVF format enables rapid qualitative detection and is the preferred option when time-to-result is critical, such as in point-of-care testing or rapid screening. On the other hand, the NALF format offers higher sensitivity, which is particularly useful for applications requiring the detection and quantification of low analyte concentrations, such as early detection of infectious diseases or environmental monitoring of contaminants. When comparing against previously reported nucleic-acid flow assays (Table S7, Supplementary Information), the biotin-MIP-based NAVF/NALF platforms presented here compare favorably in terms of operational simplicity, time-to-result, and analytical performance [15,17,38,39]. Closely related formats based on double-tagging endpoint PCR commonly rely on biological capture reagents at the test line (anti-tag antibodies or biotin–streptavidin systems) combined with nanoparticle or enzymatic readouts, typically requiring 10–20 min assay times and reporting LODs that range from ng mL^−1^ levels to pg µL^−1^ equivalents depending on the labeling and quantification approach. In contrast, the present strategy implements a robust synthetic biotin-MIP test zone and achieves rapid readout in the NAVF format (~3 min) while maintaining competitive sensitivity, whereas the NALF format reaches a quantitative LOD in the low pg mL^−1^ range using a simple enzymatic colorimetric readout with smartphone/ImageJ quantification. Although the biotin–streptavidin pair remains the gold standard in intrinsic affinity and well-characterized kinetics, performance in paper-based point-of-care devices is not governed solely by equilibrium affinity but also by practical factors such as reagent cost, batch-to-batch reproducibility, robustness to storage and handling, and stability under non-ideal environmental conditions. In this context, biotin-MIPs offer a biomimetic capture element that can be prepared in a scalable manner and integrated as a solid test zone with high chemical and thermal robustness, providing an alternative to protein-based capture layers that can be more sensitive to fabrication and storage constraints. The results show a sharply localized and stable test signal, low background (supported by PCR negative controls), and imprinting-dependent capture (supported by NIP controls), enabling competitive LODs and dynamic response ranges in both NAVF and NALF formats. Overall, these comparisons support that integrating MIPs as biomimetic test zones constitutes a practical alternative to antibody- or streptavidin-based capture in paper-based nucleic-acid diagnostics, with format-dependent advantages that can be selected according to the intended point-of-care application.

## 4. Conclusions

This work demonstrates the integration of a biotin-specific MIP as a synthetic recognition element into nucleic acid vertical flow (NAVF) and nucleic acid lateral flow (NALF) platforms for the detection of double-tagged PCR amplicons.

Although streptavidin provides the strongest known non-covalent interaction with biotin, paper-based point-of-care devices also require capture layers that are compatible with membrane processing, stable under storage and fabrication conditions, and scalable with minimal batch-to-batch variability. In this context, biotin-MIPs provide a biomimetic, animal-free alternative that can be deposited as a solid test zone within nitrocellulose while maintaining device-level analytical performance.

Both platforms showed outstanding sensitivity, with an LOD as low as 2.53 × 10^−2^ ng mL^−1^ for the NAVF and 2.95 × 10^−3^ ng mL^−1^ for the NALF. The comparative evaluation highlights the complementary strengths of the two paper-based formats. NAVF allows rapid qualitative detection within 3 min and exhibits a lower visual LOD than NALF, which is ideal in situations where immediate visual results are critical. In contrast, NALF achieves a one-order-of-magnitude improvement in sensitivity compared to NAVF and a total assay time of less than 15 min, making it more suitable for applications requiring highly sensitive semi-quantitative detection of low analyte concentrations. Accordingly, the choice between the two platforms should be guided by the specific diagnostic needs of the intended use.

The successful integration of MIPs into both conventional and non-conventional paper-based diagnostic platforms highlights their versatility and potential applicability beyond the scope of the present study. These devices also offer opportunities for further customization, allowing the detection of additional biomarkers or analytes. Although these results are promising, further validation with clinical samples and complex biological matrices is needed and will be addressed in future studies to strengthen the translational relevance of the proposed approach. Overall, this work represents a significant step forward in the development of MIP-based paper-based platforms and lays the foundation for future advances in reliable, robust, and affordable diagnostic tools. Although the need for thermal cycling may be viewed as a limitation for point-of-care deployment, recent progress in portable and compact PCR instrumentation has substantially improved the feasibility of performing nucleic acid amplification outside centralized laboratories. Portable thermocyclers, such as miniPCR [42,44] and other commercial battery-powered platforms, have shown performance comparable to conventional benchtop systems in decentralized diagnostic applications. Therefore, the paper-based MIP platform presented here is compatible with simplified molecular testing workflows based on portable PCR devices. Further integration with compact amplification hardware, and potentially with alternative isothermal amplification strategies, could enhance the practical implementation of this approach in POCT settings.

## Data Availability

The original contributions presented in this study are included in the article/Supplementary Material. Further inquiries can be directed to the corresponding author.

## Supplementary Materials

The following supporting information can be downloaded at https://www.mdpi.com/article/10.3390/bios16030175/s1: Figure S1: FT-IR spectra of biotin-MIP before and after template removal and the corresponding NIP; Figure S2: Schematic representation of the final design and layer assembly of the NAVF cartridge; Figure S3: Comparative performance of NC membranes evaluated for the NAVF; Figure S4: Specifications of the materials used to assemble different NAVF cartridges; Figure S5: Evaluation of the impact of different cartridge assemblies and spacer layer incorporation on NAVF performance Figure S6: Comparison of background noise and signal intensity obtained with C083 and CF4 cellulose-based pads; Figure S7: Evaluation of different double-sided adhesive tapes; Figure S8: Comparative performance of filter papers; Figure S9: Results obtained for the detection of double-tagged PCR amplicons of *E. coli*, using the biotin-MIP integrated into the reaction zone of the NAVF platform. Images of the triplicate cartridges for increasing amplicon concentrations, ranging from 210.8 to 0 ng mL−1 (left to right). The corresponding relative areas, obtained by processing the images with ImageJ software, are shown below each cartridge. Figure S10: Results of the NALF assay for the detection of double-tagged PCR amplicons of E. coli using the biotin-MIP as a test. Images of triplicate NALF strips for different concentrations of the amplicons, ranging from 520 to 0 ng mL−1 (left to right). The corresponding relative areas, obtained by processing the images with ImageJ software, are shown below each strip. Table S1: Sequences of primers for double-tagging amplification of *E. coli*; Table S2: Thermal cycling conditions; Table S3: Specifications of the NC membranes evaluated; Table S4: Specifications of the VF2 spacer layer; Table S5: Specifications of cellulose-based pads; Table S6: Specifications of absorbent pads. Table S7: Comparison of previously published NALF (Nucleic Acid Lateral Flow) and NAVF (Nucleic Acid Vertical Flow) platforms and signal-generation readout strategies. Reference [45] is cited in the supplementary materials.
