# Supplementary material for: Molecularly Imprinted Polymers as Biomimetic Test Zones in Paper-Based Nucleic Acid Assays—Comparing Vertical and Lateral Flow Formats
Source: Biosensors (Basel). 2026 Mar 21;16(3):175. doi: 10.3390/bios16030175 (PMC13023435; doi:10.3390/bios16030175)
Supplement: Supplementary file 1 [file biosensors-16-00175-s001.zip › biosensors-4138042-supplementary.pdf]

Supplementary Information

Molecularly Imprinted Polymers as Biomimetic Test Zones in Paper-Based Nucleic Acid Assays—Comparing Vertical and Lateral Flow Formats

Jennifer Marfà [1,2], Anaixis del Valle [1,2], Maria Del Pilar Taboada Sotomayor [3] and María Isabel Pividori [1,2,*]

[1] Grup de Sensors i Biosensors, Departament de Química, Universitat Autònoma de Barcelona,
08193 Bellaterra, Spain; jennifer.marfa@uab.cat (J.M.); anaixis.delvalle@uab.cat (A.d.V.)

[2] Biosensing and Bioanalysis Group, Institute of Biotechnology and Biomedicine, Universitat Autònoma de Barcelona, 08193 Bellaterra, Spain

[3] Department of Analytical Chemistry, Institute of Chemistry, São Paulo State University (UNESP),
Araraquara 14800-060, SP, Brazil; m.sotomayor@unesp.br

* Correspondence: isabel.pividori@uab.cat; Tel: +34-93-581-2806

S1. Instrumentation and Materials

All materials used in the construction of the NAVF device, unless otherwise specified, were supplied by Cytiva (Marlborough, MA, USA). The nitrocellulose membranes evaluated for the reaction zone included AE98 (No. 13400100), AE99 (No. 13401787), AE100 (No. 13400183), Protran BA85 (No. 10402506), and Protran BA83 (No. 13401483). Cellulose-based sample pads C083 (No. CFSP203000, Merck Millipore, Darmstadt, Germany) and CF4 (No. 8114-6621), absorbent pads CF5 (No. 8115-6621) and CF7 (No. 8117-2250), and the glass fiber spacer layer VF2 (No. 8124-6621) were also assessed. The filter papers used in this study were RM1510 (No. 1510, 60 g m^{-2}, Filtros Anoia, S.A., Barcelona, Spain), Whatman Grade 42 (No. 1442-055, Cytiva), and a thin cellulose filter paper (12.5 g m^{-2}). Double-sided adhesive tapes, including medical-grade tapes 1510 (No. 7100012004), 1522 (No. 7100009912), and 1567 (No. 7100287656), as well as microfluidic diagnostic tapes 9965 (No. 7100067080) and 9969 (No. 7100116011), were sourced from 3M (Saint Paul, Minnesota, USA).

S2. Bacterial strain, growth conditions, and DNA extraction

Escherichia coli was cultured in Luria–Bertani (LB) broth at 37 °C under aerobic conditions for 24 h until the optical density at 550 nm (OD_{550}) reached 1.0. Serial dilutions of the culture were then prepared, and 100 µL of each dilution was plated on LB agar. After incubation at 37 °C for 24 h, the concentration of viable bacteria was determined by colony-forming unit (CFU) counting and expressed as CFU mL^{-1}. For bacterial lysis and DNA extraction, 1 mL of bacterial culture was incubated at 99 °C for 10 min and centrifuged at 12,000 × *g* for 10 min. The supernatant was discarded, and the pellet was resuspended in 1 mL of Milli-Q water and centrifuged at 12,500 × *g* for 15 min. The lysate was then resuspended in 200 µL of TRIS-EDTA buffer (10 mM TRIS, 1 mM EDTA, pH 8.0) and heated at 99 °C for 15 min. After cooling on ice for 15 min, the sample was centrifuged at 12,500 × *g* for 5 min, and 10 µL of the supernatant containing genomic DNA (gDNA) was used for PCR amplification.

The primers used for double-tagging PCR were designed for the specific amplification of the 16S ribosomal gene of *Escherichia coli* strain FC5906 (NCBI Reference sequence: MN661169.1) (**Table S1**). In the proposed assay, the analytical specificity is mainly determined by the amplification step since the biotin-MIP recognizes the biotin label on the amplicon rather than the target sequence itself. Therefore, appropriate primer design and PCR optimization are essential to avoid non-specific products that could compromise assay performance. In this work, the primers were selected following standard design criteria to maximize target specificity and minimize self-

dimer, heterodimer, and hairpin formation, and the amplification conditions were optimized accordingly. Under the selected conditions, agarose gel electrophoresis showed the expected PCR product with no detectable low-molecular-weight bands attributable to primer-dimers. Furthermore, the negative control did not produce a measurable signal, which further supports the absence of relevant non-specific labelled products in the working conditions used.

Table S1. Sequences of the set of primers for the double-tagging PCR amplification of *E. coli*.

Strain	Sequence (5′–3′)	Type	5' Label
E. coli	AGAGTTTGATCCTGGCTCAG	Forward	BIO
	ATTACCGCGGCTGCTGGC	Reverse	DIG

Abbreviations: Fw, forward; *Rev*, reverse; *BIO*, biotin; *DIG*, digoxigenin.

Each PCR reaction was carried out in a final volume of 25 µL, containing 2 µL of a 1:10 dilution of the extracted gDNA, 12.5 pmol of each primer (BIO-8F and DIG-534R), and 12.5 µL of ReadyMix *Taq* PCR Reaction Mix. The thermal cycling conditions are detailed in **Table S2**. Negative control reactions, prepared without a DNA template, were included in all experiments. The resulting double-tagged amplicons were analyzed by conventional agarose gel electrophoresis to confirm correct amplification and the absence of contamination.

Table S2. Thermal cycling conditions for the double-tagging PCR amplification of *E. coli*.

	Initiation	DNA denaturation	Annealing	Elongation	Final elongation
	1 cycle		30 cycles		1 cycle
**Temperature (°C)**	94	94	58	72	72
**Time (s)**	180	30	30	45	420

S3. Characterization of molecularly imprinted polymers

S3.1 Characterization by FT-IR

FT-IR spectroscopy was used to characterize the biotin-MIP and to identify the functional groups present in the polymer network. The spectra of the biotin-MIP, before and after template removal, together with the corresponding NIP, are shown in **Figure S1**Error! Reference source not found..

The biotin-MIP exhibited peaks at 2982 and 2953 cm^{-1}, which were attributed to the asymmetric C-H stretching vibrations of methyl and methylene groups in the aliphatic polymer backbone. The presence of an intense peak at 1722 cm^{-1}, corresponding to C=O stretching vibrations of the ester

carbonyl groups, and the characteristic asymmetric and symmetric C-O-C stretching vibrations at 1252 and 1142 cm^{-1}, respectively, was consistent with the formation of a cross-linked polymer network. Additional peaks corresponding to C-H bending of $-CH_2-$ and $-CH_3$ groups, and out-of-plane C-H deformation were observed at 1452, 1387, and 756 cm^{-1}, respectively. The peak at 1637 cm^{-1}, attributed to the C=C stretching of vinyl groups, decreased significantly in the biotin-MIP after template removal and NIP, indicating a minimal presence of unreacted double bonds. Furthermore, the similar intensity of this peak in both materials suggests a comparable degree of polymerization.

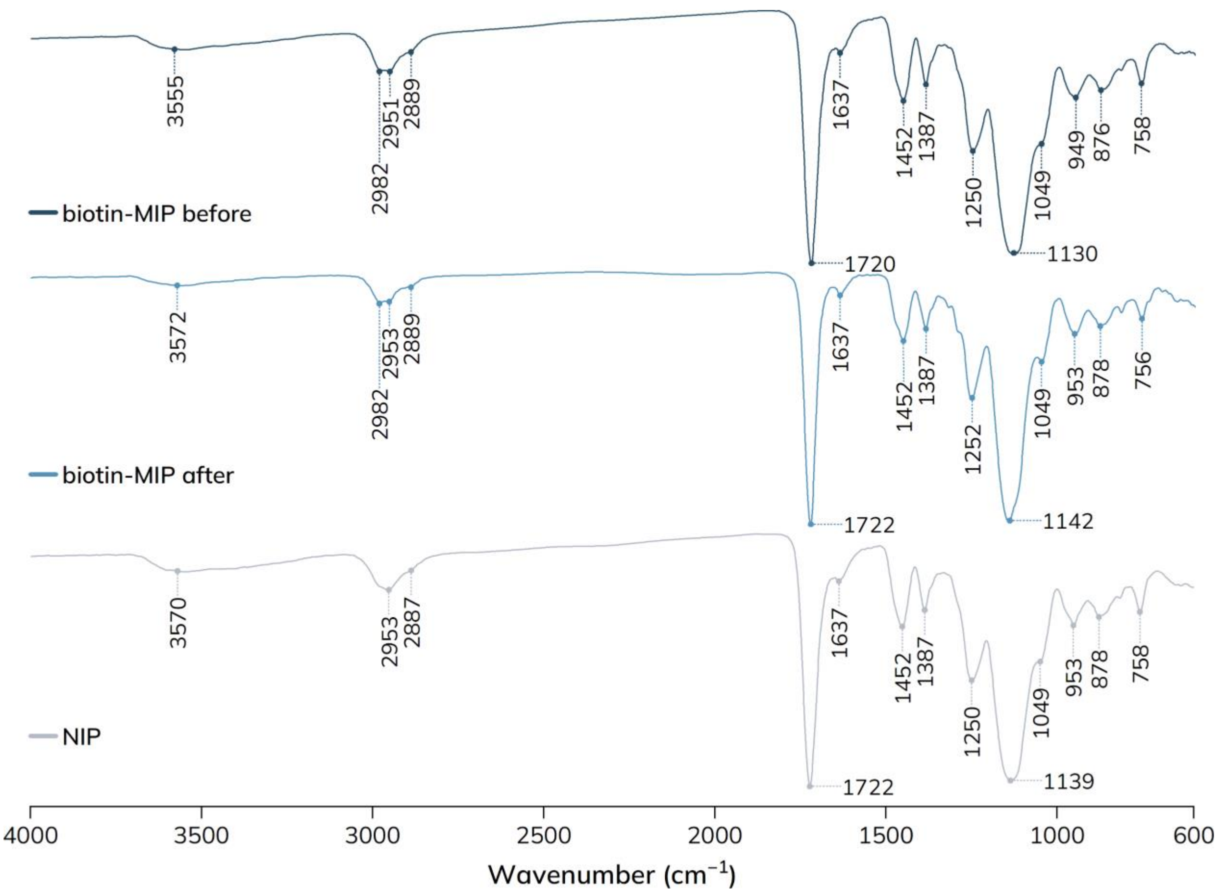

Figure S1. FT-IR spectra of the biotin-MIP before (top panel) and after (middle panel) template removal, compared to the corresponding NIP used as a control (bottom panel).

A broad band between 3600 and 3400 cm^{-1}, attributed to O-H stretching vibrations, was observed in all polymers. After template removal, this band shifted towards higher wavenumbers, which is consistent with the disruption of the hydrogen bonds previously formed between the template and the functional monomer. Importantly, the close similarity between the spectra of biotin-MIP after template removal and NIP further confirms the complete removal of the biotin template.

S4. Optimization of the integration of molecularly imprinted polymers in vertical flow cartridges

The NAVF cartridge consists of two main polypropylene components, the top and the bottom sections, which form the structural housing of the device and hold the membranes and functional layers. At the top of the cartridge, there was a plastic support (28 × 20 × 1 mm) with a 9 mm central opening. A double-sided adhesive tape of the same dimensions was applied to the backside of the plastic support to seal the central opening and ensure proper alignment of the NC membrane. All pads were laminated to 12 × 12 mm and stacked in the following order. First, the NC membrane was aligned with the central opening to ensure uniform liquid distribution. A spacer layer was placed beneath the NC membrane to maintain the necessary separation between layers and to prevent backflow. A cellulose-based pad was then included to ensure continuous sample flow through the device. To reinforce the structural integrity of the assembly, a filter paper layer (28 × 20 mm) was placed underneath. Finally, an absorbent pad (28 × 20 mm) was positioned at the bottom of the assembly to collect excess fluid. After proper alignment and placement of all layers, the bottom and top sections of the cartridge were assembled and sealed to create a compact, closed system.

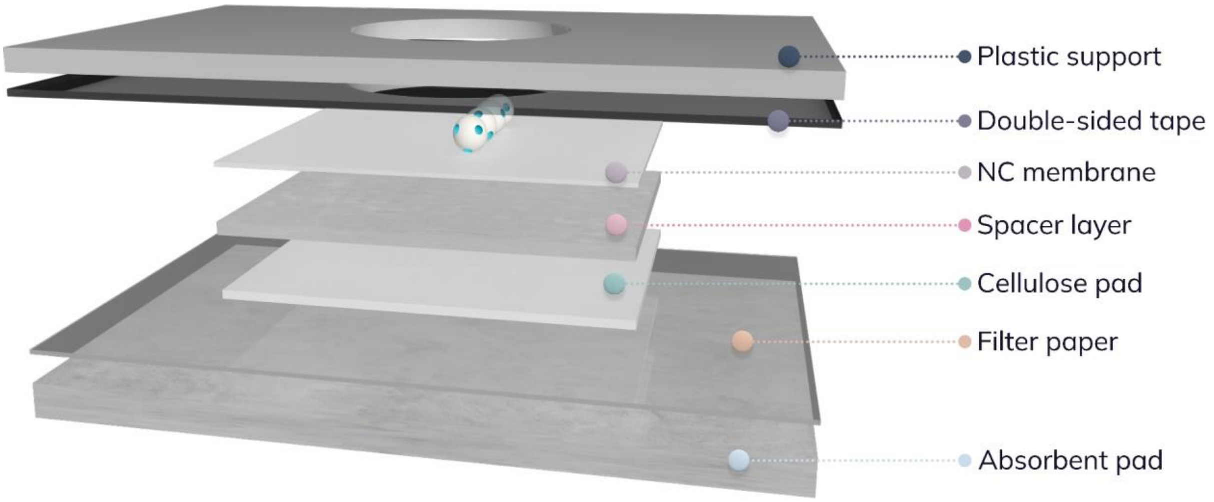

Figure S2. Schematic representation of the components used in the final design of the NAVF cartridges. From top to bottom, the assembly includes: (●) a plastic support with a central hole for sample addition; (●) double-sided adhesive tape to seal the central opening and secure all the layers; (●) an NC membrane where the biotin-MIP is immobilized; (●) a spacer layer to reduce sample retention and maintain the separation between the layers; (●) a cellulose-based pad to minimize non-specific background; (●) filter paper to reinforce structural stability and hold all stacked layers correctly assembled; and (●) an absorbent pad at the bottom of the cartridge to collect excess reagents and prevent backflow.

S4.1 Evaluation of nitrocellulose membranes

An initial screening was carried out to assess the suitability of different nitrocellulose (NC) membranes for the integration of biotin-MIP into the reaction zone of the NAVF platform. Critical

parameters considered in this study included membrane pore size, protein-binding capacity, mechanical robustness, and flow rate. The NC membranes evaluated were the AE series (AE98, AE99, and AE100) and Protran membranes BA83 and BA85. The specifications of all NC membranes evaluated in this study are summarized in **Table S3**. After deposition of biotin-MIP, the reaction zone was blocked by adding 40 µL of a 0.2% (w/v) PEG 4000 solution, followed by drying at 39 °C for 3 h. This blocking strategy effectively reduced background signals and improved the signal-to-noise ratio, particularly on the AE98 and AE100 membranes.

Table S3. Specifications of the NC membranes evaluated for the NAVF platform (AE98, AE99, AE100, Protran BA83, and Protran BA85) and the NALF strip (FF80HP).

Product	Product code	Thickness (µm @ 53 kPA)	Flow rate (s/4 cm)	Pore size (µm)
Nitrocellulose membrane				
AE98	13400100	110	160–210	5
AE99	13401787	126	120–160	8
AE100	13400183	108	90–120	12
Protran BA83	13401483	107	N/A	0.2
Protran BA85	10402506	100–160	N/A	0.45
FF80HP	10547002	200	60–100	5

The Protran BA83 and BA85 membranes showed poor performance in the NAVF configuration due to inadequate immobilization of biotin-MIP, which resulted in spreading beyond the reaction zone, low signal intensity, increased background, and irregular fluid flow. Since these limitations could not be mitigated within the assay design, Protran membranes were excluded from further studies. Among the AE membranes, AE98 showed the highest signal intensity and minimal background noise, closely followed by AE100, as shown in **Figure S3**. The pore size (5 µm) and flow rate (160–210 s/4 cm) of AE98 were particularly suitable for the NAVF format and enabled effective immobilization of the biotin-MIP. Based on its superior performance, the AE98 was selected as the reaction membrane for further development of the NAVF.

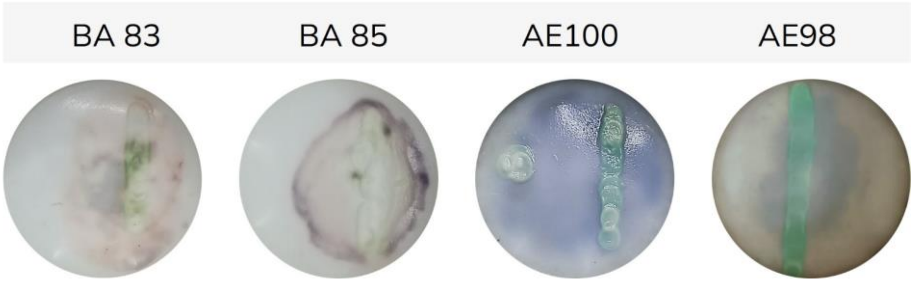

Figure S3. Comparative performance of the different NC membranes evaluated, including Protran BA83 (0.2 µm), Protran BA85 (0.45 µm), AE98 (5 µm), and AE100 (12 µm).

S4.2 *Evaluation of the spacer layer*

To ensure uniform reagent flow and minimize sample retention, a glass fiber spacer layer was incorporated between the NC membrane and the cellulose-based pad. This spacer, being inert and non-reactive toward assay reagents, promoted a faster and more uniform flow while significantly reducing sample retention compared to cotton-based materials. In addition, the VF2 spacer prevented direct contact between the NC membrane and the absorbent pad, which could otherwise lead to backflow or increased background signal.

Table S4. Specifications of the VF2 pad used as a spacer layer in the NAVF platform.

Product	Product code	Thickness (µm @ 53 kPA)	Wicking rate (s/4 cm)	Water absorption (mg/cm^2)
Spacer layer				
VF2	8124-6621	689	24	86.2

To assess the impact of the spacer layer and other device components on NAVF performance, various cartridge assemblies were designed and tested. The materials used in each configuration are summarized in **Figure S4**. In all cases, the NC membrane, double-sided tape, and absorbent pad were kept constant, and the results were qualitatively evaluated by visual inspection.

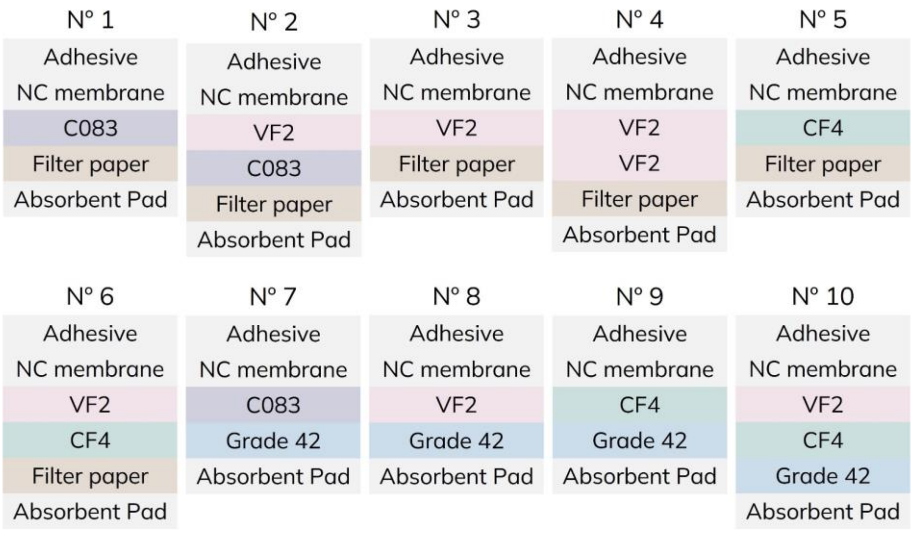

Figure S4. Overview of the materials and components used to construct the different NAVF cartridge assemblies. In all configurations, the NC membrane, double-sided adhesive tape, and absorbent pad were kept constant.

Although a high background was observed in all cases, assemblies 6, 8, and 10 demonstrated superior performance and higher signal intensity and were therefore selected for further comparison. As shown in **Figure S5**, assemblies 6 and 10, which combine the VF2 spacer with the CF4 cellulose-based pad, provided the optimal balance between rapid wicking and signal generation. Assembly 8,

combining VF2 with Grade 42 filter paper instead of the CF4 cellulose-based pad, also showed promising results, probably due to the increased thickness and higher grammage of the filter paper. However, the signal intensity obtained was lower than that observed for assemblies 6 and 10. Consequently, assembly 8 and the other tested configurations were excluded from further studies. The addition of a VF2 spacer, therefore, effectively improved assay reliability by reducing sample retention and preventing re-interaction with the upper layers. Based on these results, assemblies 6 and 10 were selected for subsequent optimization steps.

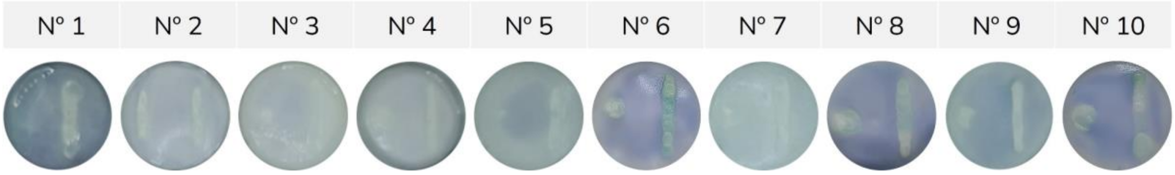

Figure S5. Results from the evaluation of the effect of the spacer layer and other device components on NAVF performance. Each image corresponds to the assembly number shown in Figure S4.

S4.3 Optimization of the cellulose-based pad

Several cellulose-based pads, including C083 and CF4, were assessed for their water absorption capacity, thickness, wicking rate, and ability to reduce background signals in the NAVF. The specifications of C083 and CF4 pads, as well as the comparative results obtained with each of them and their corresponding negative controls, are shown in **Table S5** and **Figure S6**, respectively.

Table S5. Specifications of the cellulose-based pads assessed for the NAVF platform.

Product	Product code	Thickness (µm @ 53 kPA)	Wicking rate (s/4 cm)	Water absorption (mg/cm^2)
Cellulose-based pad				
CF4	8114-6621	482	67.3	49.9
C083	CFSP203000	830	126	120

The CF4 pad demonstrated superior performance, providing a continuous and uniform flow through all stacked layers, leading to lower background signals and higher sensitivity. Its low protein-binding capacity reduced non-specific binding, while its hydrophilic properties enabled rapid re-wetting after long-term storage, maintaining functionality during extended use. Furthermore, the reduced thickness of CF4, compared with C083, promotes faster wicking without compromising signal strength, further improving both sensitivity and specificity. In contrast, although the C083 pad was thicker and had a higher absorption capacity, its lower wicking rate

resulted in higher background signals and reduced assay sensitivity. Considering all these factors, the CF4 pad was determined to be the most suitable cellulose-based pad for the NAVF platform.

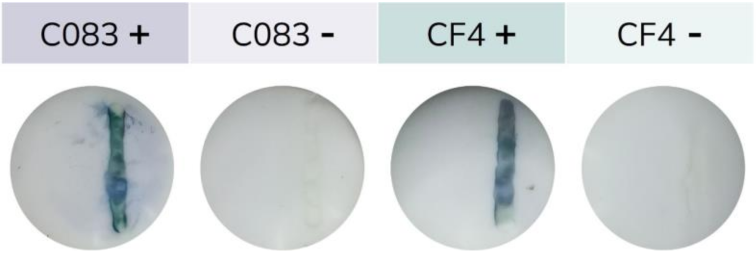

Figure S6. Comparison of background signal and signal strength obtained using C083 and CF4 cellulose-based pads, along with their corresponding negative controls.

S4.4 Evaluation of the absorbent pad

The absorbent pads, CF5 and CF7, were assessed for their capacity to handle large liquid volumes, prevent backflow, and maintain unidirectional fluid flow through the stacked membranes. An optimal absorbent pad must have sufficient capacity to retain the entire volume of assay reagents without disrupting the fluid flow or causing reagent pooling, which could lead to inconsistent results. **Table S6** summarizes the properties of the absorbent pads evaluated.

Table S6. Absorbent pad specifications used in the development of the NAVF platform, including thickness, wicking rate, and water absorption.

Product	Product Code	Thickness (µm @ 53 kPA)	Wicking rate (s/4 cm)	Water absorption (mg/cm^2)
Absorbent pad				
CF5	8115-6621	954	63.3	99.2
CF7	8117-2250	1873	35	252.3

Although CF5 (954 µm) exhibited a faster wicking rate (63.3 s/4 cm) than CF7, it also had a lower water absorption capacity (99.2 mg/cm^2) and therefore was unable to absorb the entire volume of liquid added during the NAVF procedure. In contrast, CF7, with a thickness of 1873 µm, showed a significantly higher absorption capacity (252.3 mg/cm^2) and a slower wicking rate (35 s/4 cm), which improved the sensitivity and reliability of the assay. Accordingly, CF7 was selected as the optimal absorbent pad for the construction of the NAVF device.

S4.5 Selection of adhesive and filter paper

The selection of an appropriate double-sided adhesive tape is critical for the development and optimization of the NAVF device, as it must ensure the correct immobilization of all membrane

layers while promoting a uniform downward flow. In this study, several medical-grade and microfluidic double-sided adhesive tapes (1510, 1522, 1567, 9965, and 9969) were assessed. The main selection criteria included the resistance to degradation or loss of adhesive properties upon contact with aqueous reagents, high sealing strength to prevent delamination during handling and testing, and the ability to prevent leakage while maintaining a uniform vertical flow through the reaction zone. In addition, since the NAVF format requires the use of biological samples and reagents, the adhesive must not interfere with or contaminate the assay components.

To determine the most suitable double-sided adhesive for the NAVF device, different assemblies, prepared using each adhesive tape in combination with the AE98 and AE100 membranes selected in the previous optimization steps (**Section S4.1, SI**), were evaluated. In this study, only the adhesive tape and the NC membrane were varied, while all other components of the device were kept constant. **Figure S7**, **panel A,** shows the results obtained for all the assemblies evaluated, while **panel B** presents the corresponding signal intensities obtained after processing the images using *ImageJ* software.

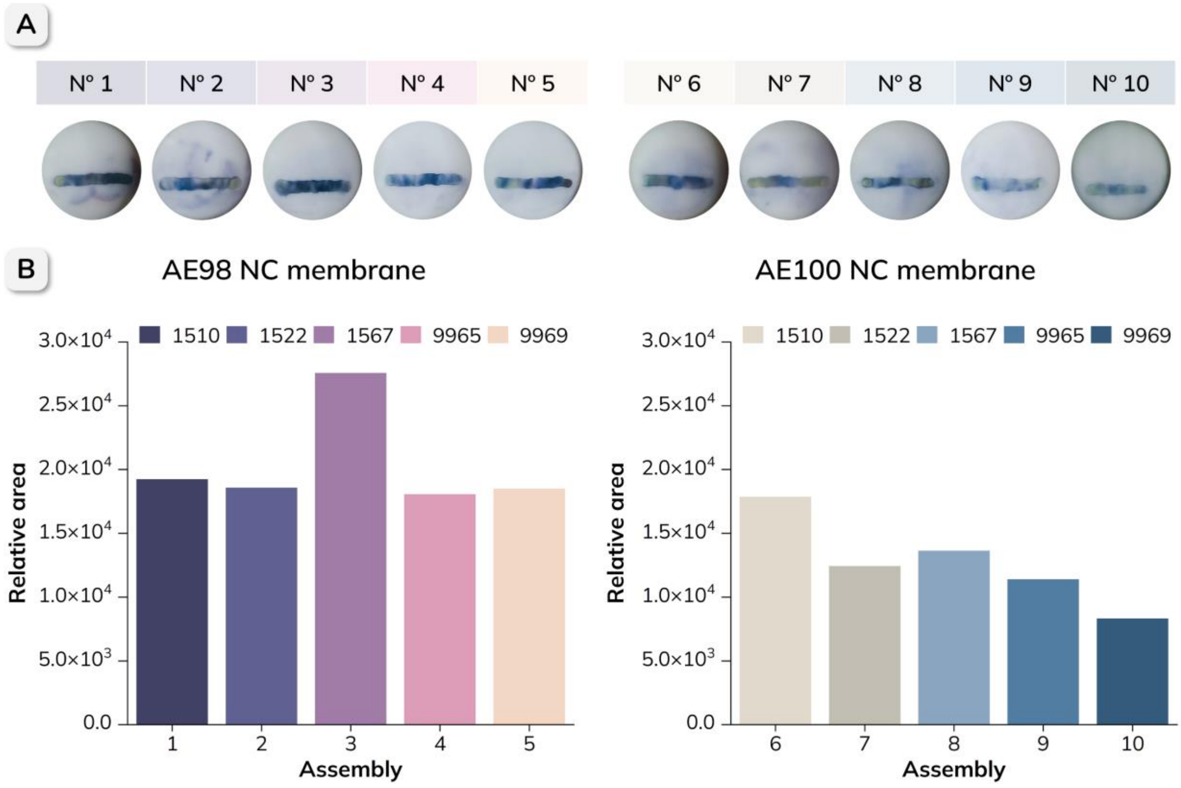

Figure S7. Evaluation of different double-sided adhesive tapes for the NAVF platform using the AE98 and AE100 NC membranes. **Panel A.** Representative images of the NAVF results for assemblies prepared with AE98 (assemblies 1–5) and AE100 (assemblies 6–10). **Panel B.** Signal intensities quantified from the images shown in panel A using *ImageJ*.

As shown in Figure S7, panel B, the AE98 membrane achieved the highest signal intensities, particularly when combined with the 1567 tape in assembly 3. In contrast, the AE100 membrane consistently resulted in lower relative signal intensities, regardless of the adhesive tape used (**Figure S7, panel B**). These results indicated that the combination of the AE98 membrane and the 1567 tape provided the best overall performance, which was attributed to the water-resistant seal of the adhesive, effective membrane immobilization, minimal reagent leakage, and consistent fluid flow.

In addition to adhesive optimization, various filter papers were evaluated to reinforce the structural integrity of the platform and to facilitate the correct alignment of all stacked layers. As shown in **Figure S8**, the use of thin filter paper (12.5 g m^{-2}) provided additional structural stability without affecting the reliability of the assay.

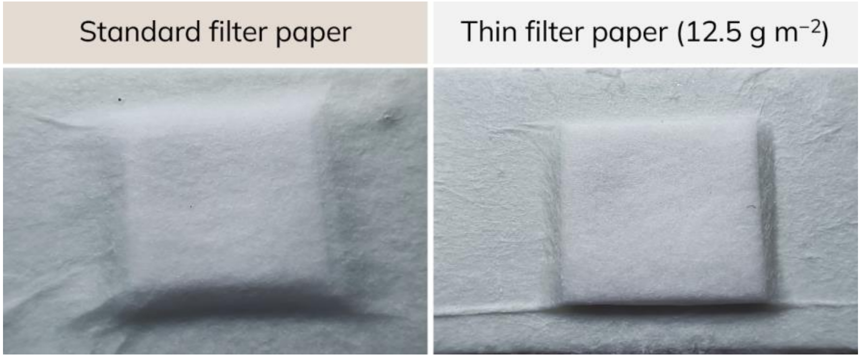

Figure S8. Comparison of standard and thin filter paper (12.5 g m^{-2}) used to reinforce the structural integrity of the NAVF cartridge.

Based on the results of the optimization studies, the NAVF cartridges were assembled using the 1567 tape, an AE98 NC membrane, a VF2 spacer, a CF4 cellulose-based pad, and a thin filter paper. This optimized final NAVF device was used for the detection of double-tagged PCR amplicons described in **Section 2.6**.

S5. Detection of double-tagged amplicons by nucleic acid vertical flow assay

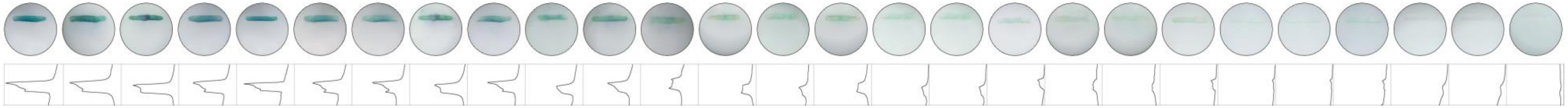

Figure S9. Results obtained for the detection of double-tagged PCR amplicons of *E. coli*, using the biotin-MIP integrated into the reaction zone of the NAVF platform. Images of the triplicate cartridges for increasing amplicon concentrations, ranging from 210.8 to 0 ng mL^{-1} (left to right). The corresponding relative areas, obtained by processing the images with *ImageJ* software, are shown below each cartridge.

S6. Detection of double-tagged amplicons by nucleic acid lateral flow assay

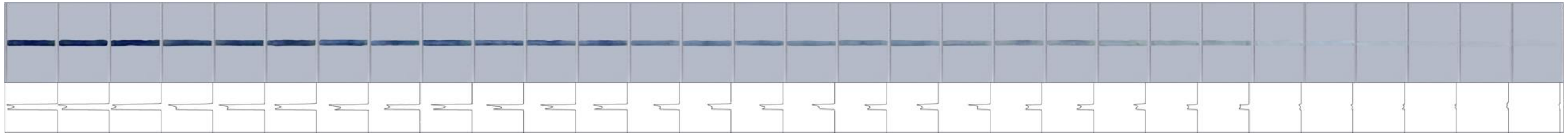

Figure S10. Results of the NALF assay for the detection of double-tagged PCR amplicons of *E. coli* using the biotin-MIP as a test line. Images of triplicate NALF strips for different concentrations of the amplicons, ranging from 520 to 0 ng mL^{-1} (left to right). The corresponding relative areas, obtained by processing the images with *ImageJ* software, are shown below each strip.

Table S7. Comparison of previously published NALF (Nucleic Acid Lateral Flow) and NAVF (Nucleic Acid Vertical Flow) platforms and signal-generation readout strategies.

Target / Analyte	Assay Format	Sample matrix	Amplification	Labels on target	Test line reagent	Signal labeling system / Readout method	Total assay time	Limit of detection (LOD)	Ref
Mycolicibacterium fortuitum	NAVF and NALF	Bacteria spiked / hemodialysis water	Immunomagnetic separation (IMS) + double-tagging endpoint PCR	BIO/DIG	antiDIG antibody	AuNP / visual and smartphone + *ImageJ* quantification	NAVF: ~1 min NALF: ~15 min	~10^2 CFU mL^{-1} (with IMS) and ~10^3 CFU mL^{-1} (without IMS)	[1]
E. coli DNA amplicons	NALF	Culture-extracted DNA amplicons	Double-tagging endpoint PCR	BIO/DIG	antiDIG antibody	CNPs and AuNPs / visual and smartphone + *ImageJ* quantification	10–15 min	CNPs: 2.2×10^{-2} pg μl^{-1} (visual) and 6.0×10^{-3} pg μl^{-1} (instrumental) AuNPs: 8.4×10^{-2} pg μl^{-1} (visual) and 2.2×10^{-2} pg μl^{-1} (instrumental)	[2]
Salmonella Enterica and *E. coli* O157:H7	NALF	Culture-extracted DNA amplicons	Quadruple-tagging endpoint PCR	BIO/FLU (*Salmonella*) BIO/DIG (*E. coli*)	antiFLU (*Salmonella*) and antiDIG (*E. coli*)	AuNPs / visual	< 15 min	7 ng mL^{-1} (*E. coli*) and 95 ng mL^{-1} (*Salmonella*)	[3]
Paracoccidioides spp. DNA amplicons	NALF	*Paracoccidioides brasiliensis* extract from clinical samples	Double-tagging endpoint PCR	BIO/DIG	antiDIG antibody	CNPs / visual and smartphone + *ImageJ* quantification	20 min	0.21 ng (visual) and 0.10 ng (instrumental)	[4]
E. coli DNA amplicons and Group B *Streptococcus*	NALF	Clinical vagino-rectal swab samples	Double-tagging endpoint PCR (portable thermocycler)	BIO/DIG	Biotin-MIP	antiDIG–HRP + TMB (enzymatic colorimetric) / visual and smartphone + *ImageJ* quantification	10 min	2 ng mL^{-1} (visual) and 1.8 ng mL^{-1} (instrumental)	[5]
E. coli DNA amplicons	NAVF and NALF	Culture-extracted DNA amplicons	Double-tagging endpoint PCR	BIO/DIG	Biotin-MIP	antiDIG–HRP + TMB (enzymatic colorimetric) / visual and smartphone + *ImageJ* quantification	NAVF: ~3 min NALF: ~15 min	NAVF: 1.0×10^{-2} ng μl^{-1} (visual) and 2.53×10^{-2} ng μl^{-1} (instrumental) NALF: 3.17×10^{-2} ng μl^{-1} (visual) and 2.95×10^{-3} ng μl^{-1} (instrumental)	**This work**

References

1. Ben Aissa, A.; Araújo, B.; Julián, E.; Zanoni, M.V.B.; Pividori, M.I. Immunomagnetic Separation Improves the Detection of Mycobacteria by Paper-Based Lateral and Vertical Flow Immunochromatographic Assays. *Sensors* **2021**, *21*, 5992, doi:10.3390/S21185992/S1.

2. Porras, J.C.; Bernuz, M.; Marfa, J.; Pallares-rusiñol, A.; Martí, M.; Pividori, M.I. Comparative Study of Gold and Carbon Nanoparticles in Nucleic Acid Lateral Flow Assay. *Nanomaterials* **2021**, *11*, 1–11, doi:10.3390/nano11030741.

3. Ben Aissa, A.; Jara, J.J.; Sebastián, R.M.; Vallribera, A.; Campoy, S.; Pividori, M.I. Comparing Nucleic Acid Lateral Flow and Electrochemical Genosensing for the Simultaneous Detection of Foodborne Pathogens. *Biosens. Bioelectron.* **2017**, *88*, 265–272, doi:10.1016/j.bios.2016.08.046.

4. Mussin, J.; Giusiano, G.; Porras, J.C.; Corredor Sanguña, L.H.; Pividori, M.I. Carbon Nanoparticle–Based Lateral Flow Assay for the Detection of Specific Double-Tagged DNA Amplicons of Paracoccidioides Spp. *Microchim. Acta* **2024**, *191*, doi:10.1007/s00604-024-06367-4.

5. Marfà, J.; del Valle, A.; Rovatti Pupin, R.; Baro, B.; Bassat, Q.; Taboada Sotomayor, M.D.P.; Pividori, M.I. Biotin-Specific Molecularly Imprinted Polymers as a Biomimetic Test Line in Lateral Flow Assays. *Biosens. Bioelectron.* **2026**, *298*, 118415, doi:10.1016/j.bios.2026.118415.
